# On pickles: biological and sociocultural links between fermented foods and the human gut microbiome

**DOI:** 10.1186/s13002-021-00458-9

**Published:** 2021-06-09

**Authors:** Andrew Flachs, Joseph D. Orkin

**Affiliations:** 1 Department of Anthropology, Purdue University, West Lafayette, IN USA; 2 Institut de Biologia Evolutiva, Universitat Pompeu Fabra-CSIC, Barcelona, Spain

**Keywords:** Ethnozymology, Fermentation, Heritage, Metagenomics, Probiotics

## Abstract

**Background:**

The composition of the human microbiome varies considerably in diversity and density across communities as a function of the foods we eat and the places we live. While all foods contain microbes, humans directly shape this microbial ecology through fermentation. Fermented foods are produced from microbial reactions that depend on local environmental conditions, fermentation practices, and the manner in which foods are prepared and consumed. These interactions are of special interest to ethnobiologists because they link investigations of how people shape and know the world around them to local knowledge, food traditions, local flora, and microbial taxa.

**Methods:**

In this manuscript, we report on data collected at a fermentation revivalist workshop in Tennessee. To ask how fermentation traditions are learned and influence macro and micro ecologies, we conducted interviews with eleven people and participated in a four-day craft fermentation workshop. We also collected 46 fermented food products and 46 stool samples from workshop participants eating those fermented foods.

**Results:**

We identified ten major themes comprised of 29 sub-themes drawn from 326 marked codes in the transcripts. In combination, this analysis allowed us to summarize key experiences with fermentation, particularly those related to a sense of authenticity, place, health, and the discovery of tactile work. From the 605 amplicon sequence variants (ASVs) shared between food and fecal samples, we identified 25 candidate ASVs that are suspected to have been transmitted from fermented food samples to the gut microbiomes of the workshop participants. Our results indicate that many of the foods prepared and consumed during the workshop were rich sources of probiotic microbes.

**Conclusions:**

By combining these qualitative social and quantitative microbiological data, we suggest that variation in culturally informed fermentation practices introduces variation in bacterial flora even among very similar foods, and that these food products can influence gut microbial ecology.

## Background

Human beings teem with billions of microbes, a complex assemblage of viruses, bacteria, and eukaryotes that together encompass a human microbiome. Ethnobiologists have long recognized that situated communities of local ecological knowledge and practice shape variable landscapes and build cultural identity in conversation with those landscapes [1–3]. While ethnobiologists are quick to recognize managed landscapes like farm fields or agroforests as shaped by sociopolitical and historical forces, we have been slower to recognize how the same processes might affect microbial landscapes including our own bodies. However, studies on the human microbiome are clear that these microbial ecologies are influenced by the places where we live and the foods we ingest [4, 5]. Microbiological evidence increasingly demonstrates that our kitchens and gardens affect our microbial ecology in dramatic and complex ways [6] because humans domesticate species, change habitats, and process foods in ways that have distinctive effects on microbial communities in our homes, our foods, and our guts.

While all foods contain microbes, humans seek out microorganisms through fermentation. By intentionally shaping microbial ecologies, we have an added layer of practice, heritage, and knowledge that influences the microbial communities we consume. Scholars are already thinking through ethnozymology [7], the ethnobiology of fermentation, by looking to the unique notions of taste, opportunities for sharing foods, and cultivation of distinctive plants, animals, and microbes made possible through fermentation and local food traditions [8–12]. As a field with longstanding interest in experiences of health, heritage, and holistic ecosystem functioning, ethnobiology has a unique perspective on how ethnozymological food culture sustains unique communities of microbes alongside sociocultural wellbeing. This geographic variation is of special interest to ethnobiologists who study knowledge, practices, and ecologies bound to place.

Fermentation brings together many topics of interest to ethnobiologists [13], including health, local ecological management, place- and practice-based knowledge, qualitative experiences with the natural world, and the advocacy of local food sovereignty in the face of an increasingly homogenized [14] global food system. Anthropologists and evolutionary biologists have shown how humans shape landscapes and domesticate species in ways that benefit their wellbeing while simultaneously creating a new set of ecological parameters that structure human and environmental possibilities [15, 16]. The same dynamics occur within the human body, where human health, food production, and local food cultures shape and are shaped by the microbial worlds we share. Fermentation is a useful heuristic to link these topics as it draws attention to craft food-making, taste and identity, and the transmission of knowledge.

In this paper, we report on qualitative anthropological and quantitative metagenomic data that describe links between fermented food production, fermented food consumption, and human gut microbial ecology. By combining qualitative participant observation and interviewing during a fermentation revivalist workshop in 2018 with metagenomic analysis of fermented foods and human stool samples created before, during, and after the workshop, we show that the microbial landscapes produced during fermentation are highly variable and can be grouped by type, as well as that microbes fostered during fermentation interact with human gut ecologies. While subtle variations in preparation and ingredients can affect distinct microbiological landscapes present in fermented foods, those microbial communities also cluster by food type. Our analysis also suggests that specific foods serve as vehicles for potentially probiotic, as well as neutral and potentially harmful, bacteria. Further, qualitative data suggest that respondents develop strong personal relationships with these foods and use fermented foods to cultivate new kinds of relationships with people, environments, and their own bodies. Our combination of anthropological, microbiological, and genetic approaches presents an opportunity to understand how sociocultural conceptions of heritage and wellbeing influence microbial landscapes in foods and in human bodies, ultimately in ways that may have health and socioecological consequences.

### Merging microbiology and social science through the ethnobiology of fermentation

An ethnobiological approach to fermentation and the microbiome opens a dialogue between microbiology and social science. Much of this conversation has centered around microorganisms’ role in human metabolic function, the political implications of seeing microbes as enemies, neutral parties, or allies, and the ways in which human existence is made plural through microbial entanglement [17]. Given this contemporary moment of *microbiomania* [18] across the social and natural sciences, scholars are increasingly asking how socioecological practices including growing, preparing, and consuming fermented foods, sustain microbial communities, heritage foodways, and human health.

A range of biomedical research has shown how human gut microbiomes shift in response to environmental context. Such studies have found significant differences between small-scale rural and industrialized urban populations in Papua New Guinea [19], Russia [20], Burkina Faso [21], Tanzania, [5, 22], and in the Venezuelan Amazon and rural Malawai [23]. In addition to these comparative studies, other research suggests that the same human population can alter its gut microbiome composition by moving. Migrants who come from rural areas in Asia to the USA experienced a loss in microbiome diversity [24], while Hadza gut flora can shift seasonally as a function of foods available and consumed [22].

Such studies evaluate microorganisms with respect to their ability to alter gut ecology in ways that correct non-biodiverse or otherwise unhealthy bodies. Noting how this governance over “right” and “wrong” bodies is reminiscent of Foucauldian biopolitics, Paxson [25–27] identifies a “microbiopolitics” inscribed in food safety regulations and everyday governance over human-microbe relationships in personal hygiene. Louis Pasteur’s germ theory and the fear that unhealthy citizens might spread disease through dangerous microbes inspired a range of state hygiene regulations, the biopolitical culmination of a world in which microbes are inherently dangerous and destabilizing to human wellbeing [27, 28]. As fermentation involves the intentional introduction of microbes, Paxson and others [18, 29] argue that fermenters occupy a post-Pasteurian ontological position in which some microbes may be dangerous, but others are helpful or neutral. Such microbial entanglements provide new ways for people to reframe microbial discourse away from the inherently perilous threats to bodies imagined by Pasteur and toward a world of promising interconnection [30, 31].

In reimagining relationships between people and microbes, Paxson contends that we can also reimagine relationships between people and institutions. Haraway [32] and Tsing [33] similarly leverage the post-Pastuerian logic of fermentation and composting to imagine communal transformation in the face of socioeconomic collapse. Following a collapse and genocide in ex-Yugoslavia, Jasarevic [34] argues that Bosnian kombucha starters help citizens begin to repair ethnonational distrust through microbial gifts and creative reorganizations of bacteria and yeast. Promising interconnection can lead to commercialization in the artisanal wine and kombucha industries, where scholars ask how producers, consumers, and regulators imagine microbiology in the context of food safety, consumer desire, and state oversight. In practice, these industries demand a microbiological citizenship in which stakeholders respect microbial beings as intentional agents that require extra-legal negotiations between producers and consumers [29, 35]. Where these scholars emphasize the present and future, some biological anthropologists also use microbes to imagine the past. Noting that pasteurization would not have been present in the paleoenvironmental context of *Homo sapiens* evolution, Speth [36] argues that the regulatory push to exclude microbes from contemporary industrial food systems, including the consumption of fermented foods, now contributes to dietary mismatches that negatively influence contemporary health outcomes. Other microbiome-curious paleoanthropologists investigate the opposite direction, analyzing the microbiota in preserved dental films to reconstruct past diets and ecologies [37, 38].

Land, states, soil, and microbes all meet in fermentation, where local socioecological variations in recipe and autochthonous bacteria transform vegetables and dairy products into distinctive foods. Much foodways scholarship demonstrates how foods are used to stake ethnic and other identity claims [39–41]. Fermented foods are not subtle. The microbially rich worlds of fermented foods allow communities to stake identity claims that are both phenomenological, through the pungent sensations of fermented foods, and microbiological: Korean food scientists evoke microbes to argue that Kimchi is uniquely Korean and not Japanese [42]; far-right Hindu nationalists make claims to native microbiota from native cows in Indian soils to envision a pure, native agricultural state [43]; indigenous Siberian communities reclaim fermented meats to distinguish themselves from Russian assimilation [44]; communities hail Bulgarian yogurt [45, 46] or Ghanaian dawa-dawa [47] because they claim that these provide unique health benefits through unique *Lactobacillus* bacteria in the face of globalizing food systems. Beyond staking ethnic or community claims, fermentation builds new and imagined communities because ferments are live cultures and can be shared through active starter cultures and brines. Like Maussian gifts, they carry the spirit of the giver alongside particular tastes and sociopolitical meanings when shared [34, 48]. To share and consume these foods is to embrace wider relationships.

This perspective from food studies suggests that microbes in fermented foods and in bodies intersect with sociocultural notions of identity. Biologically, diet in general [49] and fermented food products in particular have been shown to influence the composition of human microbiomes [50], a point seized by Danone Yogurt’s nutritional research team [51]. However, determining the exact links between particular food products, including fermented foods is difficult outside of clinical settings. Fermented foods vary widely in the microbes they might introduce to the human microbiome because these foods are eaten as part of larger food systems and because a number of confounding sociocultural factors determining how people eat complicate how people ultimately encounter microbes. While private companies or local advocacy groups might promote the nutraceutical benefits of fermented foods [12, 52], ethnobiologists recognize as well that these foods rely on in-situ conservation of microbial and agricultural biodiversity [7, 8, 10], and folk microbiology to continue practicing and adapting this social food system.

Ethnobiologists interested in how fermented foods become part of larger food systems have looked to fermentation in the contexts of food sovereignty and food security. Ethnobotanists ask how fermentation secures access to taste and nutrition in the Balkans [7, 53] and across Europe generally [11, 12], while others track how wild plants introduce autochthonous bacteria that makes fermented beverages in the archaeological record [54, 55] and in the present [56, 57]. Fermentation is also emerging as a means for traditional knowledge revival, as when communities frustrated with the offerings of global agrifood networks turn to fermented food traditions to seek out distinctive tastes [25, 58, 59] or rediscover heritage recipes [60]. Through recipe-sharing networks, gift exchanges of microbial cultures [34], or workshops like the one profiled below, fermentation revivalists are working to conserve and cultivate local and heritage biodiversity down to the microbial level. Where private companies and regulatory systems grapple with a new politics of food safety [25, 29, 61], ethnobiologists are increasingly recognizing ways in which agrobiodiversity, traditional knowledge, and microbial diversity might go hand in hand [62].

## Methods

### Our mixed-methods approach

This scholarship suggests two key questions that emerge from an attempt to combine cultural anthropology and microbiology in the study of ethnozymology. First, do the local and heritage recipes that give fermented foods their distinctive flavors result in measurable differences in the resulting food products? That is, is one sauerkraut microbiologically distinct from another in ways that have impacts on the human microbiome? Teams including the Benjamin Wolfe lab [63, 64] have been asking similar questions by inoculating gnotobiotic (microbe-free) cabbages to observe the results, while Marcellino et al. [65] examined differences in bacterial genera from French cheeses of differing regions. Although the resulting ecosystems appear to be similar with respect to microbial composition, cheese and sauerkraut microbes vary as a function of carbon assimilation and salt tolerance. This suggests that minor differences in craft process have profound impacts in what fermented food lovers perceive as taste. In an agricultural riff on microbial research, *terroir*, the sociocultural value of local environmental conditions in food, is here reimagined as a quantifiable local microbial ecology. Where ethnobiologists [8] and anthropologists [26] emphasize how microbes accumulate monetary value when artisanal practices are recognized as a microbial *terroir*, microbiologists [61, 65, 66] are tracking how aggregated culinary and landscape management decisions, ranging from wine barrels to cheese caves, affect microbial ecology in measurable and reproducible ways. These studies suggest that social and microbial cultures can be linked in productive ways.

Second, do microbial communities present in fermented foods influence the human microbiome in ways that have longstanding or beneficial effects? This is the argument made by researchers affiliated with the probiotics industry, such as the Danone Yogurt research team. This same kind of claim must also be regulated under a microbiopolitics that lends state support to microbial health. However, few studies track these interactions outside of clinical settings, in the spaces where people live and eat. Overwhelmingly, health and diet scholarship on fermentation and the microbiome is dominated by clinical or genetic approaches. One recent report with wide-ranging sociocultural implications on the influence of migration and dietary change on human microbiomes in the USA even employs community-based participatory research methods to collect its data, but relies entirely on quantitative and biomedical analysis to explain the relationship between migration, microbiome, food, and health [24]. While emphasizing the importance of subtle distinctions in place, conceptions of health, and dietary practices, these studies do not incorporate sociocultural qualitative data into their analysis. This is a missed opportunity because this research assumes but does not investigate the importance of local knowledge vested in communities that shape dynamic ecosystems and create possibilities for future socioecological relationships.

These are, fundamentally, sociocultural questions that have to do with how people live and eat. They represent the structuring conditions under which the microbial landscape of a fermented food and a human gut microbiome interact. To begin answering both questions, we conducted interviews, participant observation, and recipe and process elicitation in combination with the collection of biological samples of fermented foods and human stools at a 2018 workshop led by fermentation revivalist Sandor Katz [48, 67]. Katz runs a biannual 4-day fermentation workshop for experienced food fermenters at his homestead in Liberty, Tennessee. As the event asked participants to pay according to a sliding scale, we recognize that this particular group and workshop is not necessarily representative of all fermentation revivalists, let alone the broad and diverse population who regularly consumes fermented foods around the world. However, their experiences and diverse histories with fermentation and larger bodies of heritage foodways comprise a wide set of interesting perspectives. Given the hyper-local nature of microbiome research, even this small group can help to illuminate our larger research questions into the synergies between life experiences, place, and microbiome.

As social scientists who study biological and cultural diversity, we wanted to learn about the traditions that sustain microbial communities through fermentation. This led to a research design including both qualitative ethnographic elements as well as quantitative microbiological elements. Along with ten other fermentation enthusiasts, including school teachers, business owners, food professionals, and students, we learned to make microbially rich foods including kimchi, sake, tempeh, and yogurt. Drawing on the opportunities of event ethnography [68], in which a small team participates in and collects a variety of data from a larger event, we drew on the accessibility of this workshop and the eagerness of event participants to share information, speak with each other, discuss nuances in shared ideas, and develop new ways of approaching group and individual decisions. Andrew Flachs, a cultural anthropologist, held interviews with all consenting participants, recorded parts of the workshop, convened group discussions on cooking, fermenting, and heritage foods, asked participants to list recipes and outline social networks related to fermented foods, and kept field notes detailing participation and observation in the workshop. Joseph Orkin, an anthropologically trained geneticist, collected biological samples of the fermented foods at the workshop, including yogurt, pickled vegetables, kimchi, sauerkraut, and miso. To collect gut microbial data that we could then associate with the lived experiences of the study participants, we asked each of them to collect a stool sample each day over the duration of the workshop, and then to mail a stool sample 1 week after the workshop ended. Through this design, we combined methods from metagenomics, computational biology, and cultural anthropology to seek to conduct a holistic analysis of the linkages between fermented foods, local environment, and public health.

#### Qualitative interview methodology

In total, we were able to speak with and participate alongside eleven individuals (six used he/his pronouns and five used she/hers pronouns). We collected 16 h of recorded conversation and interviews, in addition to workshop field notes and photography. We transcribed these data and coded them using the qualitative data analysis software QDAMiner to draw out both responses to specific research questions around recipes, heritage, and knowledge revitalization, as well as inductive themes developed through a grounded theory approach [69]. In total, we identified ten major themes comprised of 29 sub-themes drawn from 326 marked codes in the transcripts. In combination, this analysis allowed us to summarize key experiences with fermentation, particularly those related to a sense of authenticity, place, health, and the discovery of tactile work. To keep personal identities and potential health data anonymous, we use pseudonyms in this and other articles. We additionally assigned color codes to each individual’s sample and dissociated identifiable personal information from the microbial analysis. This way, no genetic analyst, including Orkin, has access to any identifiable information from workshop participants, and no qualitative data analyst, including Flachs, has access to the raw genetic data associated with human stools. We feel it is important to report this, as best practices will likely change in the future, and our intention and responsibility is always to the rights to privacy of our research interlocutors.

#### Food and gut microbiota sampling methodology

We collected a variety of food samples produced and consumed during the 4-day fermentation workshop (Table 1). After collection, we stored all samples (food and fecal) at – 80 °C at Purdue University. We collected each food sample using gloved hands and sterile tools. Liquids were collected with disposable sterile pipettes and solids with disposable chopsticks covered with sterile 1-mL pipette tips. In each case, 1-mL samples were placed into sterile 2-mL Eppendorf tubes and immediately frozen in liquid nitrogen to arrest microbial growth. We collected 46 fermented food samples, which we grouped into 5 main categories based on their primary composition: beans, dairy, grains, salted vegetables, and sugared vegetables (Table 1). Within these categories, we sampled 13 different types of ferments and 20 food items. For 11 of these food items, including rapid-fermenting foods like tempeh and kefir chèvre, we were able to collect samples from multiple time points throughout the fermentation process. Our initial intent had been to collect samples from beginning, middle, and end fermentation points of each food item. While we were largely successful in this effort, the nature of our participant observation in the course did not always allow for this. In cases for which the length of time required to complete a ferment for typical consumption extended beyond the length of the course, we sample the latest time point possible prior to our departure. Some types of fermentation have encompassed different food items (e.g., multiple varieties of koji-based fermentation).

**Table 1:** Fermented foods collected during the fermentation course. Fermentation time categories are listed as “Finished” if active fermentation was halted prior to consumption; those samples listed as “End” were not fermented to completion, but are the last chronological sample we collected within a food item. “Cumulative hours fermented” represent the total time that a food item was fermented. For example, the koji rice sake was fermented for 25.5 h beyond the koji rice that was used as an ingredient. An * indicates fa ood samples for which we were not able to determine an exact count of hours fermented

Sample ID	Food category	Ferment	Food item	Fermentation time category	Hours fermented	Cumulative hours fermented
**SK001**	Bean	Acaraje	Acaraje	Finished	*	*
**SK002**		Dosa	Dosa	Finished	24	24
**SK003**	Dairy	Goat Milk	Goat milk	Start	0	0
**SK011**		Kefir	Kefir grain	Starter	Starter culture	Starter culture
**SK006**			Kefir chèvre	Start	0	0
**SK008**				Early	8	8
**SK009**				Late (curd)	24	26.5
**SK010**				Late (whey)	24	26.5
**SK007**				Finished (curd)	26.5	26.5
**SK044**		Yogurt	Yogurt	Start	0	0
**SK045**				Finished	8	8
**SK050**	Grain	Buckwheat batter	Buckwheat batter	Finished	*	*
**SK004**		Injera	Injera	Early	3	3
**SK005**				Finished	48	48
**SK015**		Koji	Koji barley	Start	0	0
**SK017**				Early	8	8
**SK016**				Finished	24	24
**SK018**			Koji barley miso	Start	0	31
**SK019**				Early	8	43
**SK022**			Koji rice	Start	0	0
**SK024**				Middle	8	8
**SK023**				Late	24	24
**SK025**				Finished	34	34
**SK031**			Koji rice sake	Start	0	34
**SK033**				End	25.5	59.5
**SK035**			Koji rice shio	Start	0	34
**SK036**			Koji rice shoyu	Start	0	34
**SK034**		Salt rise bread	Salt rise bread	Finished	~ 24*	~ 24*
**SK041**		Tempeh	Tempeh	Start	0	0
**SK043**				Early	8	8
**SK042**				Finished	24	24
**SK012**	Salted vegetables	Kimchi	Kimchi	Start	0	0
**SK014**				Early	8	8
**SK013**				End	24	24
**SK028**		Pao cai	Pao cai	Early	0	8
**SK026**				Early	8	8
**SK027**				Early	8	8
**SK030**				Early	8	16
**SK029**				End	24	32
**SK067**		Sauerkraut	Sauerkraut	End (brine)	*	*
**SK065**				End (cabbage)	*	*
**SK037**	Sugared vegetables	Sweet potato fly	Sweet potato fly	Start	0	0
**SK040**				Early	17	17
**SK038**				Middle	24.5	24.5
**SK039**				Late	36	36

Fecal samples were collected daily by each participant beginning on the first morning of the course. Each participant defecated into a garbage bag placed over the toilet seat and with gloved hands used disposable chopsticks covered with sterile 1 mL pipette tips to place ~ 500 mL of feces into sterile 5 mL Eppendorf tubes prefilled with 400 mL of 100% ethanol. Fecal samples were labelled with the collection date and unique color codes then stored at room temperature. Upon conclusion of the course, we gave each participant a package containing these collection materials and prepaid shipping envelope to collect a fifth fecal sample (using the same technique) one week after the course concluded. We chose to collect fecal samples in ethanol rather than liquid nitrogen in order to ensure methodological consistency between fecal samples collected during and after the course. In total, we collected 46 fecal samples from 11 people. From seven of them, we received samples on each of the five requested days. Two participants gave us samples on each of the four days of the course, but did not return the final sample. Two individuals provided only one or two samples, and they were removed from the analysis.

#### Molecular and computational biology methodology

All molecular lab work was conducted by the University of Minnesota Genomics Center. Food and fecal DNA was extracted using the Qiagen DNeasy Powersoil HTP kit with bead beating and extraction blanks. For each DNA sample, we generated 16S v4–v5 amplicon libraries using a 2-step dual-indexing polymerase chain reaction (PCR) approach [70]. We pooled all samples for sequencing with a single run of an Illumina MiSeq with 2 × 300 V3 paired-end chemistry.

In order to identify the unique microbial taxa in our samples, we generated amplicon sequence variants (ASVs) from the 16S sequencing reads using the DADA2 pipeline [71]. To avoid spurious results, we excluded from analysis any sample with fewer than 1500 reads. We removed sequencing adapters with cutadapt (Martin, 2011), identified optimal trimming parameters with FIGARO [72], and assigned taxonomy with SILVA nr_v138 [73]. Microbial community analysis was conducted in R using the Phyloseq package [74]. We removed reads that were classified as belonging to unknown phyla, cyanobacteria, or mitochondria. To identify the presence of distinct microbial communities among food and fecal samples, we calculated Bray–Curtis beta diversities using relative abundances, which we ordinated with nonmetric multidimensional scaling (NMDS). Given the variety of collected samples, we sought to minimize the contribution of rare taxa by filtering our dataset to exclude ASVs not present at least five times in any sample. We also removed samples containing fewer than 2000 sequencing reads to minimize spurious results. To test for significantly distinct microbial communities, we conducted PERMANOVA tests with 9999 permutations.

We sought to identify bacteria that could have been transmitted from fermented foods to the gut microbiome of workshop participants. To minimize the risk of mistakenly identifying contaminants, we excluded any ASV that was identified in a control sample, and removed samples with fewer than 2000 reads. However, we retained low-abundance OTUs given the exploratory nature of this analysis. We then normalized microbial abundances with DESeq2 [74, 75]. We subset our data to only include microbes that were present in at least one food sample and the fecal sample taken on the final day of the workshop, but not present in any fecal sample collected on the first day. Finally, we removed any ASV with a higher mean normalized abundance in the food samples than the fecal samples.

## Results and discussion

### Sociocultural themes in the experience of fermenting

Our goal in collecting qualitative anthropological data was to relate the stories and the practice that fermentation revivalists carry with them to the microbiology we would detect later. From this perspective, every family recipe and kitchen improvisation is a step toward shaping microbial biodiversity in our foods and in our own bodies. Every cheese cave, wine valley, and cabbage patch likely has its own collection of microbes, to say nothing of our kitchens and stirring spoons. After transcribing interviews and uploading field notes into QDAMiner, we identified a number of inductive and deductive themes that we will explore below.

The workshop was a masterclass in learning to cultivate biodiversity through smell, taste, and texture, well suited to the anthropological methods of participant observation and open-ended interviewing. Beginning with a sweeping introduction on the history of fermented foods Katz oscillated between microbiological discussions of specific strains, proteins, and fermented foods, and observations rooted in sensory perceptions and general guidelines. Although we discussed how *Lactobacillus* species tolerate salty and acidic environments in ways that other bacteria do not, Katz quickly lightened the mood as we nervously asked if we were preparing the food correctly. “When I make a brine, I like it to taste like the sea,” he offered, inviting us to taste, rather than measure our way to a successful microbial ecology. The following day, he continued to first situate us within scientific concepts, such as a chemical description of anaerobic respiration, before emphasizing “but today we’re going to learn and get more of the experience – what it smells like, what it feels like.” These shifts between the scientific nomenclature and phenomenological ways of knowing centered the group’s expectations somewhere between culinary and biochemical expertise.

The workshop created an educational space apart from microbiopolitical food safety regulation, catering to an educated group of fermenters eager to similarly claim expertise within a range of sensory, scientific, and culinary bodies of knowledge. Unsurprisingly, as Katz’s bookshelf featured anthropological work including Paxson’s [25] *The life of cheese*, Katz and workshop participants rejected the Pasteurian logic of food regulation in favor of actively shaping a microbial landscape that was influenced by a larger socioecological landscape. During the first day, several participants asked how best to avoid harmful bacteria or contamination, questions motivated by living within the larger US and Canadian regulatory system that polices bacteria. Explicitly influenced by Paxson, Katz deconstructed these assumptions, explaining “there’s not a registry of good and bad bacteria…we ultimately don’t know enough about microorganisms to make these moral judgements.” Rather, he described fermentation as a means for shaping a particular community through different pushes and pulls of acidity, vegetable matter, and salinity. “Of all the communities of microorganisms, the question is which will develop?” he continued. Conceived as such, the practice of fermentation is thus a practice of creating different landscapes, what Katz called “manipulating environmental conditions” to make a fermented food amenable to acidic, salty, hot, or ammonium-rich bacterial communities.

Workshop participants seized on this heuristic of shaping environments as they reflected on their experiences with fermentation during interviews and participant observation. Maya, a trained cook and fermentation entrepreneur, spoke specifically about interacting with microbial environments. “I don’t really do anything,” she explained, contrasting her experience as a high-end chef to her new work as a fermented food seller decentered from the creation of her products. “I mean, I’m not making the vegetables ferment. I’m just creating an environment where that will happen. You know, I’ve learned along the way certain things to make sure that I have a better result, but I’m not actually fermenting things…I set it all up and I step out of the way.” No longer a cook as such, Maya describes her work in terms of being an environmental steward. Carl, a Midwestern IT professional, similarly referenced letting the fermented environment work to cultivate unique, strong flavors at the microbial level that are otherwise absent in the larger food system. “We can introduce a lot of umami flavors, it seems like…that’s part of the funk that develops,” he explained. This search for distinctive tastes differs from the nationalist discourses around fermentation described above in that Carl is not recreating family or place-based recipes so much as chasing the byproducts of this microbial ecology. “In all these ferments there’s the souring that happens through the production of lactic acid. Some people can taste the difference between lactic acid and acetic acid, and I can, and I prefer the lactic acid.” Carl, who distinguishes between the vinegary taste of acetic acid and the sharper “funk” of lactic acid, takes pride in the ways that he has learned to shape the distinctive variations in his food. Although he references protein structures and glutamate compounds in our discussion, Carl’s definition of a successful ferment leans more on sensory experiences of taste and touch to guide the ferments that he creates.

While all the fermenters referenced microbes in our interviews, most described fermentation as part of a larger set of environmental practices including gardening, foraging, or simply walking in forested spaces. Maya, who grew up eating fermented food, began making fermented products after learning to have “a conversation with plants. And that was so exciting for me to, like, meet the neighborhood go around and spend a lot of time on the trails…and then as a chef you get knowledge of plants and you’re like ‘Okay – how do I eat you?’ Or, what medicine are you. What’s your power, what’s your personality?” Similarly, Karen, in the early stages of starting a fermentation business, explained “I started looking at how I could value the marginal, like the weeds in my garden. Instead of ripping them out. And so we started playing around with dandelion, and nasturtium, and calendula marigold, and infusing those into our krauts…So we thought between the wild fermentation and the wild edibles, lets market ourselves as this business that helps you re-wild yourself and get back in touch with nature.” Every member of the workshop took pride in gardening or growing foods as a way to resist homogenizations of flavor or in the experience of eating. Maya’s idea of meeting the neighborhood and Karen’s hope that fermentation could help her family and her customers “re-wild” themselves speaks to the larger ways in which fermentation is helping this community embrace a place-based knowledge and praxis. All but two fermenters mentioned that fermentation has entered their lives along with small-scale vegetable cultivation and foraging, particularly growing cabbage that can be used for sauerkraut or kimchi.

Workshop participants described how these small changes to the landscapes where they live influenced their fermentation, while Katz reminded us they are part of the story of heritage fermentation as well. A few hours into the workshop, Katz drew our attention to the ecological constraints within a larger landscape that would influence the possibilities for a microbial landscape. “You know the famous Belgian open fermented beers,” he told us. “Where that happens is in a valley that was once dominated by cherry orchards. So if you have a concentration of a certain kind of agriculture in the geographical area that would certainly have implications for the density of microbes in the air, the distribution of what kind of microbes you find in the air…and then I think that that’s definitely an aspect of *terroir* that people are talking about more and more.” Where some commercial food suppliers leverage local microbes as a scientific marker of *terroir* [26, 61], the participants in this workshop saw microbial *terroir* as a means to an end featuring creativity, flavor, and distinction in an alternative food system. “The first time I made kraut I remember moving my body,” explained one participant, “squeezing the fruits, the vegetables, it’s like a work out in a really nice way…the physical component of it is just really important to me to feel more connected to the food.” *Terroir* as such is a means to an end, not a marker of high-end cuisine or geographic distinction in a market but a result of a locally situated authenticity. As Paxson and Helmreich [31] argue, microbes in this context provide a way to think through larger sociopolitical relationships between people, place, and the food system. In particular, fermentation as a skill rediscovered or reaffirmed in spaces like these workshops help to support a body of knowledge and practice where participants feel greater control and agency over the foods they eat and the spaces where they live. Kelsey, who came to fermentation after seeking to rebuild her gut microbiota, sees the creative possibilities of learning to ferment as a means to build ecological knowledge. “It’s amazing that we’re in this period of time when people can reach adulthood with such a limited skillset, or zero skillset,” she laments. Her enthusiasm for fermentation has led her to both new foods and new medicines as she explores non-pharmaceutical and plant-based medical traditions.

Similarly, several participants credited fermentation with providing a new kind of creative outlet through which they could both experiment and actively reject a homogenous food system. “With the work that I do, it is kind of nice cooking. I mean, because even an act as simple as just slicing up cabbage can feel, like: well here’s what I’ve done,” said Matt, a pastor from Pennsylvania. “There’s just some immediate satisfaction when others can join you and then you can gather around a table. It’s really nice.” Warren, a professor at a liberal arts college who had joined him for the drive to Tennessee, described this process as riffing. “Initially I’ll look at a recipe and follow it, but I’m always riffing on it…what I'm feeling spontaneously in the moment.” While providing him with a creative outlet, the improvisatory nature of fermentation also helped him embrace more diverse food options. “The whole “Walmartization and McDonaldization of society, especially in the West that we live in. It’s creeping in everywhere, right? I mean the homogeneity, it’s just dumb and it takes away the beauty of a difference, right?” During a later interview, another fermenter was more direct: “I feel a little bit constitutionally unable to *exactly* follow a recipe.” We laughed, but there may be something about encouraging bacteria to grow on your food that attracts people hostile to the industrial food system.

Warren was not alone in these sentiments. Several other participants and Katz himself contrasted the diversity of flavors and knowledge of local and fermented foods with a larger homogenization in a global food system. Signaling cooperative and anti-establishment politics in American Left and Green circles [39], the workshop space featured counter-culture books, zines, and artwork lamenting corporate control in industrialized agriculture. Like Warren and Kelsey, Katz placed the practice of fermentation within a larger critique of a Pasteurian state that welcomed industrialized agriculture and criminalized local and microbially variable food production. “I feel like, here we are among this population of de-skilled, infantilized people, and you know food is just food – it’s this transaction at the supermarket. And depending on how much money you have in your wallet you have more choices, but I just feel like food is so much deeper than that.” It is beyond the scope of this paper to review the scholarly literature on the ways in which food is and is not a capitalist commodity [76–79], but ethnobiologists and agrarian studies scholars recognize the transformative effects of practice on knowledge and identity [80, 81]. Fermenters were keenly aware of the need for knowledge-sharing spaces like this workshop to structure the transfer of knowledge and microbial cultures. While all in attendance had read at least some of Katz’s books early in their experience, most mentioned informal networks that allowed them to swap recipes, find community, and, importantly, share microbially rich cultures that could be used as starters for fermented foods like yogurt, sourdough bread, or kombucha. As living gifts, these fermented starters share qualities with seeds, or plant starts studied by ethnobiologists [34, 82], sustaining not just a food product but also a social relationship.

In addition to distinctive carriers of place, flavor, and skill, several fermenters used the workshop to embrace the healing potential of a balanced microbial ecosystem. Four told stories in which fermented foods were central to their experiences of living with chronic illnesses, including cancer, HIV, and severe gastrointestinal distress. Warren, a cancer survivor, explained that he first encountered fermented foods by looking through peer-reviewed literature on probiotics, particularly kombucha. “Yeah when the cancer thing happened, I'm the type of person where I’m kinda all in. Alright, okay, let’s fight this right?” Later, he continued, “I probably drink 7-14 ounces at least per day.” Fermenters who looked to these foods for health did so within a larger dietary context of promoting some kind of healthy gut flora in their own bodies. Although many had read medical reports on the microbiome, none articulated specific probiotic or prebiotic genera that they hoped to introduce into their bodies. Rather, they came to imagine some microbes as allies in their fight to stay healthy and others as impediments. “I know we can’t put it into good and bad like that, but you can turn a dis-biotic situation into a balanced situation,” argued Kelsey. “I probably took antibiotics, the five day Z packs [azithromycin], twice a year every year,” she continued. Kelsey blames this childhood exposure to antibiotics for chronic bowel problems, a condition that led her first to read Weston Price’s [83] early work on comparative nutrition and later to literature on fermentation and the microbiome. Like Warren, she pursues fermentation not necessarily because it provides a specific healthy bacterial genera but because she wants to create a healthier environment within herself where her gut flora can flourish. “So you arrived to adulthood and you have these problems but it's not mysterious…it's like going to battle, you kill first and then you have to regenerate. And I did that with bone broth and with fermented foods.” Both Kelsey and Warren used military language to describe how specific fermented foods provided microbial allies for their bodily ecosystems, noting their frustration with conventional medical approaches that favored chemotherapy and antibiotics, respectively. More generally, fermenters named benefits including clearer skin, regular bowel movements, stronger immunity to seasonal illnesses, and improved energy throughout the day. “There’s literally a quickening, you feel yourself coming alive from the inside out. And then you start to seek those foods out,” explained Maya. Coming alive, which Warren also described, is a function of how fermenters imagine themselves to be reshaping their bodies through the live microbial cultures of their fermented foods.

Both implicitly and explicitly, the creative and engaged changes that fermenters experienced as they learned to make fermented foods then manifest in how fermenters imagine themselves to cultivate the microbial communities within themselves and their foods. Over the course of our interviews and participation in the workshop, the fermenters discussed questions of health, place, and creative labor on scales ranging from fermenting crocks to farms. In agrarian studies and ethnobiology scholarship [1, 13, 84], landscapes are assumed to be in dialectic relationship with the cultures that manage them. There are no farms without farmers, and local cultural knowledge or norms relating to that natural resource management goes on to create new habitats for human and non-human life. However, the innumerable dietary and lived confounds in the human body, as well as the difficulty in studying how fermented foods impact the gut microbiome in the places where they live and eat mean that we cannot assume that the same dynamics work the same way in our fermented foods. To test what sorts of microbial ecologies fermenters created when they made these foods, as well as how those ecologies interacted with human bodies, we conducted a series of microbiological and genomic analyses.

### Food and gut microbial analysis: results

**Fig. 1:**
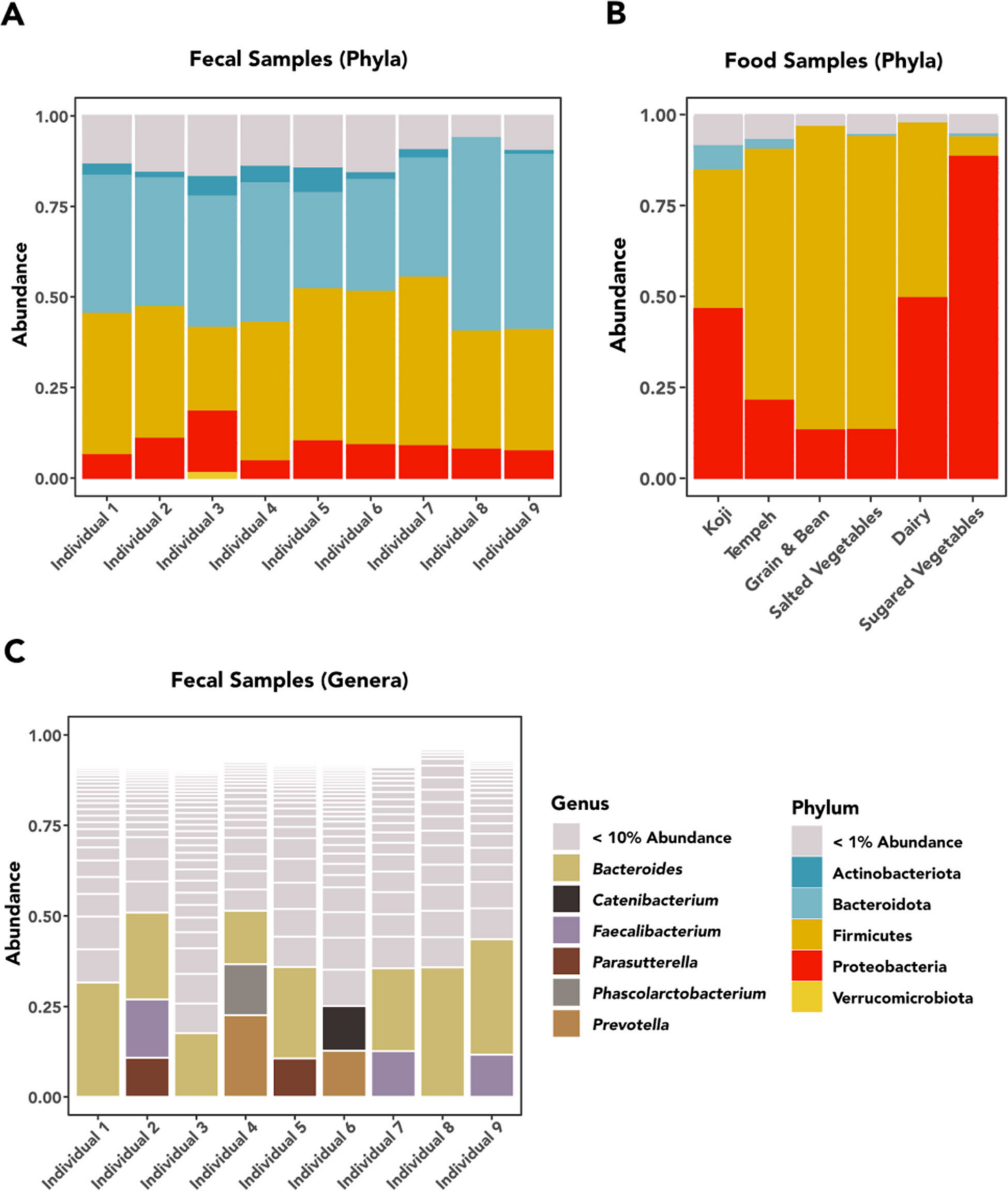
Relative abundance of microbiota in food and fecal samples

**Fig. 2:**
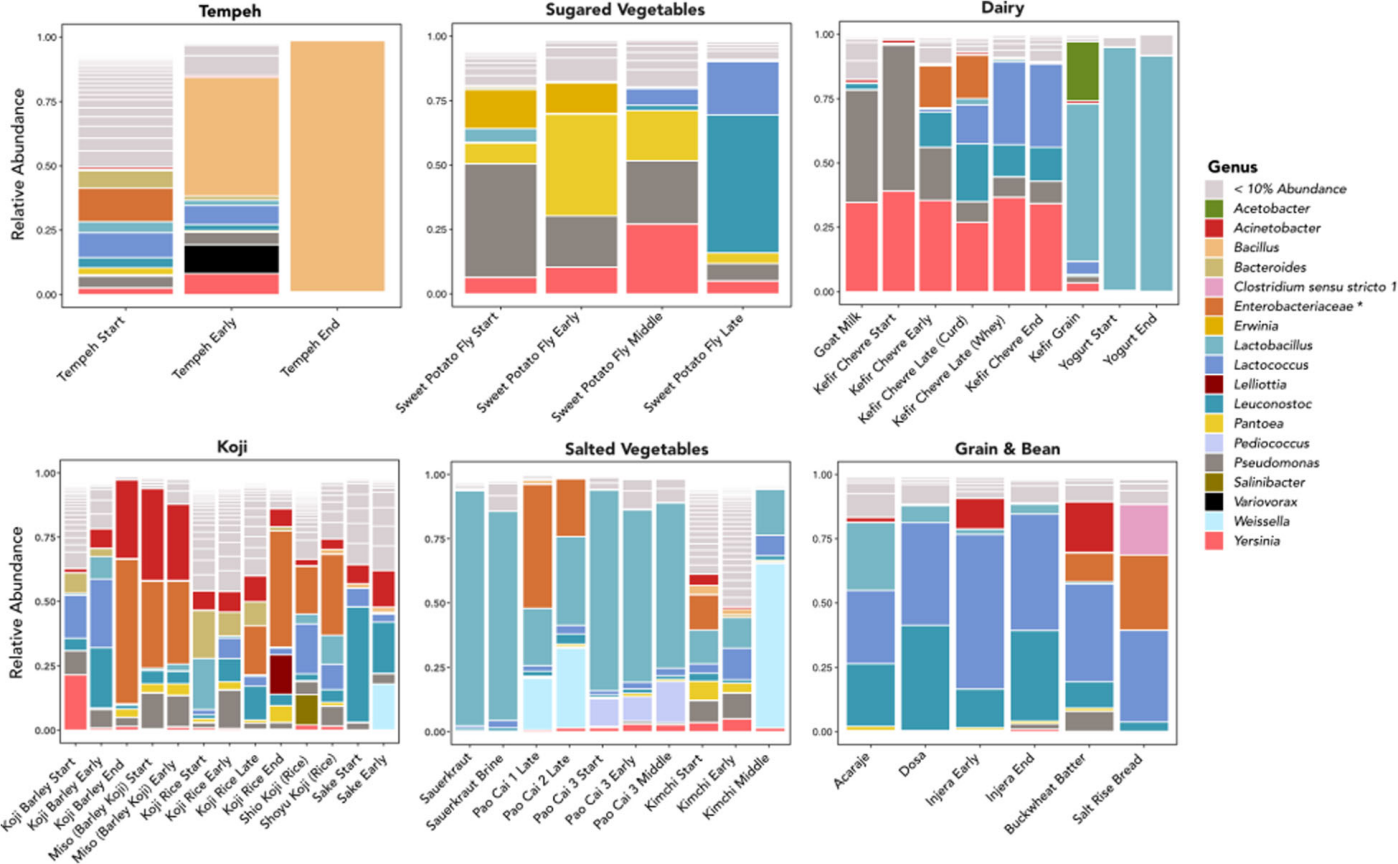
Relative abundance of bacteria genera in fermented food samples. Genera shown are those present with at least 10% relative abundance in at least one food sample. Genera comprising less than 10% abundance are represented in light gray to improve visualization for comparison. Salted vegetables, wild fermented grains, beans, yogurt, sake, and the kefir grain are dominated by lactic acid fermenting bacteria (shades of blue). Enterobacteriaceae* comprises a single high-abundance ASV that could not be resolved at the genus level

In total, we identified 6923 ASVs from the 82 samples that passed quality control. The 40 food samples contained 1595 ASVs, and we identified 6067 ASVs in 42 fecal samples. Of the 6923 ASVs, 605 were shared between the food and fecal datasets. Both food and fecal samples were dominated by a small number of phyla. The fecal samples were dominated by Bacteroidota, Firmicutes, with lesser contributions of Proteobacteria, Actinobacteria, and low-abundance phyla (Fig. 1a). The food samples contained predominantly members of the Firmicutes and Proteobacteria (Fig. 1b). At the generic level, most food samples were represented predominantly by a small number of high-abundance genera, predominantly lactic acid bacteria and Gammaproteobacteria (*Lactobacillus*, *Lactococcus*, *Leuconostoc*, *Pseudomonas*, *Weisella*, *Yersinia*, *Bacillus*, *Acinetobacter*, and *Pantoea*) (Fig. 2). In total, we identified 226 different variants of lactic acid bacteria in our food samples, 90 of which belong to the genus *Lactobacillus*. In contrast, the microbial communities of the fecal samples generally were less dominated by high-abundance genera, with *Bacteroides*, *Faecalibacterium*, *Parasuterella*, *Phascolarctobacterium*, *Prevotella*, and *Catenibacterium* being the most common taxa (Fig. 2).

**Fig. 3:**
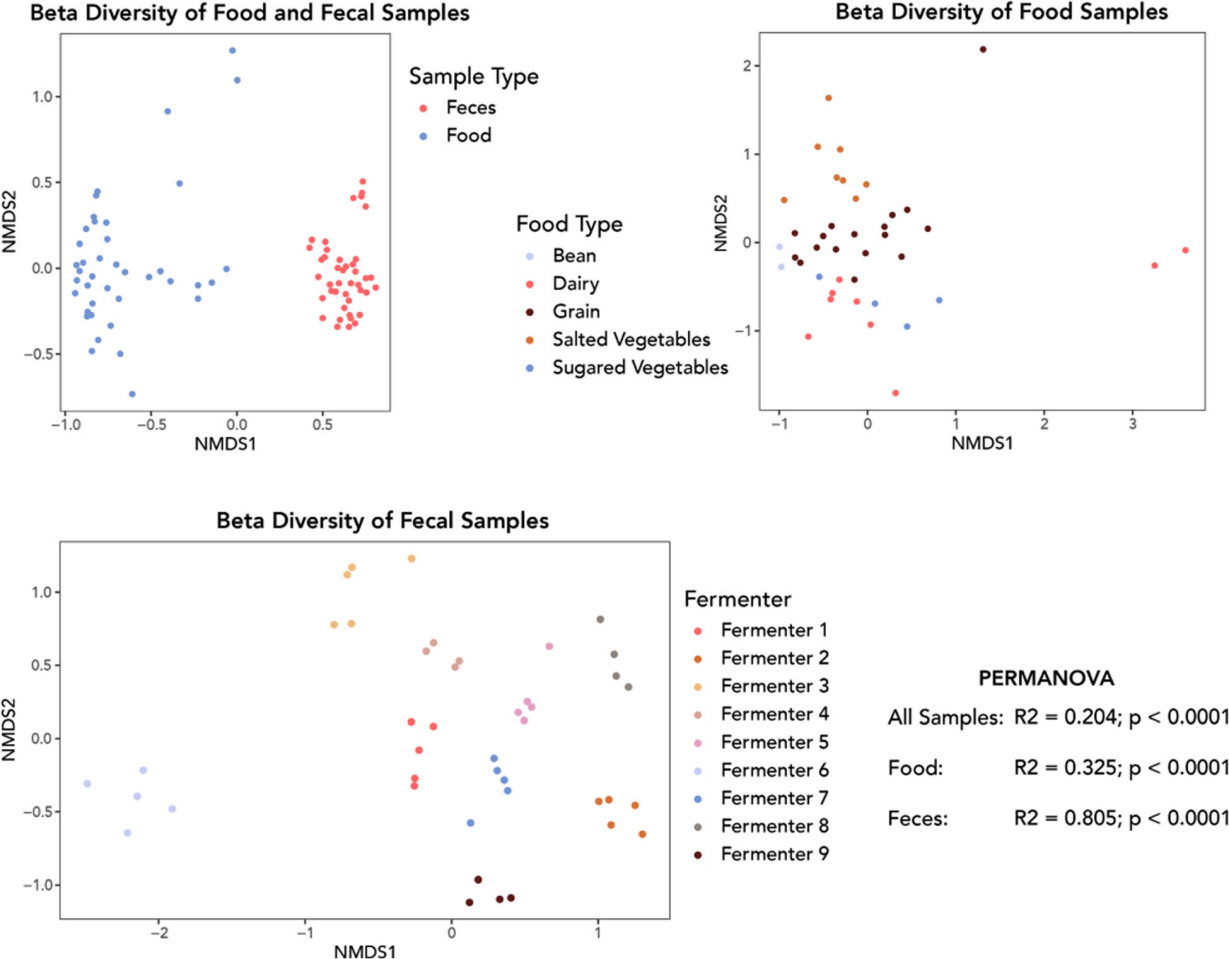
Bray–Curtis beta diversity ordinations of food and fecal samples

Beta diversity ordinations indicated a clear and significant separation (PERMANOVA: *R*^2^ = 0.204; *p* < 0.0001) in the food and fecal microbial communities (Fig. 3, top left) along the first dimension. Food samples clustered into distinct communities (PERMANOVA: *R*^2^ = 0.325; *p* < 0.0001) corresponding with the five food categories listed in Table 1 (Fig. 3, top right). The final tempeh sample (24-h fermentation) and the two yogurt samples plot as outliers from the other members of their corresponding food categories. Bacterial communities within the fecal samples (Fig. 3, bottom) clustered tightly by individual (PERMANOVA: *R*^2^ = 0.805; *p* < 0.0001). The chronological order of the fecal samples was not significantly associated with fecal beta diversity (*p* = 0.197). Along the first dimension there was a clear separation between one individual, Fermenter 6, and the other members of the workshop, and in general, beta diversity was evenly spread along the second dimension. This separation may be explained by a difference in relative fermentation experience or medications taken between Fermenter 6 and the others.

From the 605 ASVs shared between food and fecal samples, we identified 25 candidate ASVs that are suspected to have been transmitted from fermented food samples to the gut microbiomes of the workshop participants (Table 2). Seven of 25 ASVs were from the genus *Lactobacillus*, followed by 2 each of *Bacillus*, *Lactococcus*, *Leuconostoc*, and single representatives of 12 other genera. In three cases, we were able to identify these ASVs to the species level (*Bacillus cereus*, *Lactobacillus paracasei*, and *Lactobacillus curvatus*).

**Table 2:**
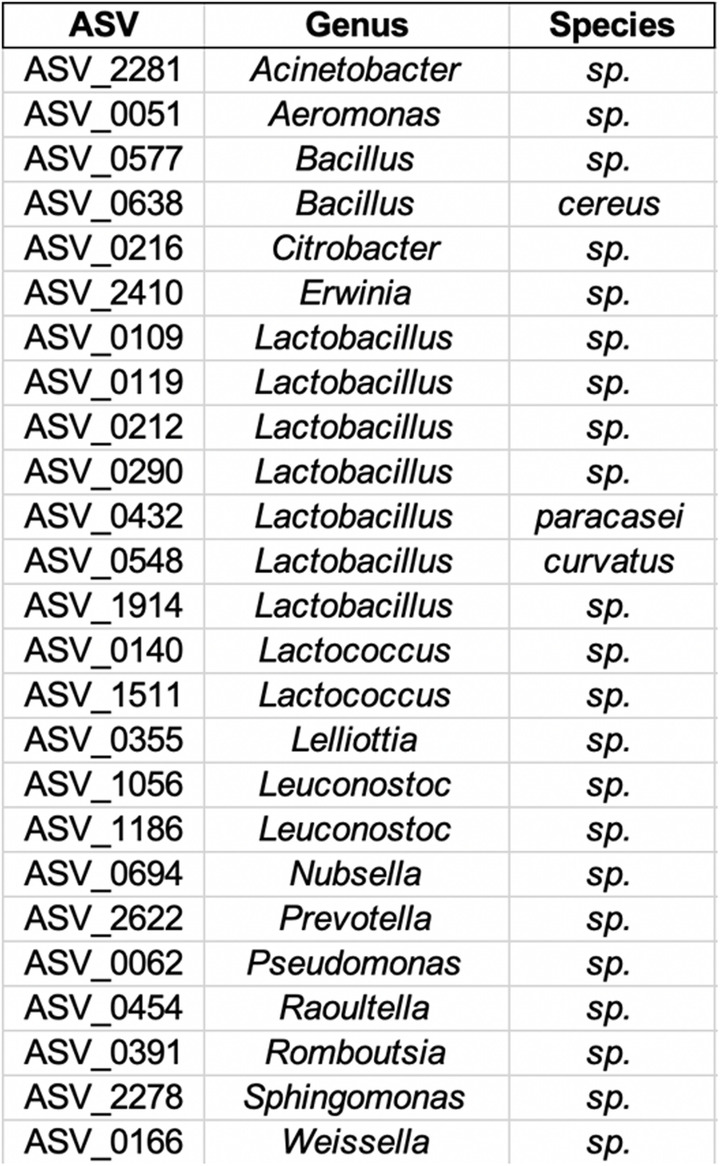
ASVs identifiable to the genus level that are suspected to have transmitted from fermented foods to the gut microbiomes of fermentation workshop participants

### Food and gut microbial analysis: discussion

#### Bacterial diversity and variation

As expected, the microbial abundance and diversity found in the fecal and food samples indicate distinct communities. We identified far more bacteria in the fecal samples and stark differences in community structure. Whereas the food samples were often dominated by a small number of taxa, the fecal microbial communities were more diverse. Among the fecal samples we observed a clear pattern of clustering by individual, which indicates that while the broader gut microbial communities of the participants fluctuated throughout the workshop, diet alone was not a dominant determinant of composition over this time period. In contrast, the fermented food samples largely clustered according to their broader food category (e.g., grain, salted vegetable), although the amount of time that a food fermented had a strong influence on the microbial communities. As expected, the fermentation process had a substantial effect on the microbial communities of many foods in relatively short periods of time. Additionally, we did observe some evidence to indicate that bacteria originating in the food samples were transmitted to the guts of the workshop participants.

To paraphrase a point that Katz made early in the workshop, fermentation is the transformative action of microorganisms, and desirable changes are subjective and culturally determined. Throughout the fermentation process we observed substantial changes in microbial communities that coincided with increased fermentation time. In some cases, there was a stark transition from a highly diverse to a nearly uniform community. After 24 h, once the tempeh had become completely covered in white mold, the bacterial community was almost entirely composed of the genus *Bacillus*. In similar fashion, the early-stage kimchi microbial community reflected a high number of low-abundance genera, but after a full day of fermentation, it came to be dominated by lactic acid bacteria, predominantly *Weissella* and *Lactobacillus*. In other cases, some microbes persisted throughout fermentation in spite of a large scale turnover. The early- and middle-stage sweet potato fly was dominated by Gammaproteobacteria (*Pseudomonas*, *Yersinia*, and *Panotoea*), but after ~ 36 h of fermentation, they were overtaken by lactic acid bacteria. The kefir chèvre bacterial community began as very similar to that of the goat milk (predominantly *Yersinia* and *Pseudomonas*). However, as fermentation continued, *Pseudomonas* was largely replaced by lactic acid bacteria (*Lactococcus* and *Leuconostoc*), while the *Yersinia* persisted. Curiously, *Lactobacillus* and *Acetobacter* were the predominant bacteria genera in the kefir grain, but these genera do not appear to have transferred to the kefir in large numbers. In contrast, the yogurt we produced originated from the same goat milk as the kefir chèvre, but the microbial community was almost entirely *Lactobacillus* at the start and finish of fermentation. This is likely a result of the milk being heat treated prior to its inoculation with the yogurt culture killing most of the microbes that originated in the goat milk.

We also observed some evidence that subtle changes in preparation can bring about major differences in microbial communities. For example, we used the same koji starter to inoculate both cooked rice and barley, and while both had similar bacterial communities by the end of fermentation, subsequent culinary processes had strikingly different effects. We added cooked mashed garbanzo beans to the barley koji to begin the transformation into miso. The early-stage miso saw an increase in the abundance of *Pseudomonas*, but the bacterial community was not largely altered otherwise. In contrast, the addition of salt, soy sauce, and wild fermented rice to produce shio koji, shoyu koji, and sake, respectively resulted in substantial increases of different lactic acid bacteria. We also observed subtle variation in our pao cai samples. We sampled two batches of Katz’s pao cai, a mix of vegetables with Sichuan spices, salt and sugar, that had been fermenting for one week (pao cai 1) and six months (pao cai 2). Pao cai 3, made during the workshop, was a mixture of the two preexisting brines with the addition of fresh vegetables. The bacterial genera in the older two pao cai were largely the same, but differed in relative abundances; this could reflect differences in age or the seasonings used. In contrast, pao cai 3 lacked the high abundance of Enterobacteraceae ASV found in the other samples and had a strong presence of an additional lactic acid bacteria, *Pediococcus*. Each of the three pao cai were eaten by workshop participants and differed slightly in flavor and texture. Given the continued replenishment of vegetables and seasonings, this finding suggests that pao cai could be a particularly variable source of probiotic microbes.

#### Pathogens, probiotics, and microbial transmission

In many cases, the predominant microbial genera that we identified in the fermented food samples were lactic acid-fermenting bacteria, such as *Lactobacillus*, *Lactococcus*, *Leuconostoc*, *Pediococcus*, and *Weisella*. This was the case for bean-based ferments, salted vegetables, yogurt, some of the grain-based ferments (salt-rise bread, injera, and buckwheat batter). This result is consistent with the widespread appreciation of the role of such microbes in fermentation, and their probiotic effects [48, 85, 86]. In some cases, we also observed other probiotic bacteria. For example, *Clostridium butyricum* was present in several ferments, most notably in salt-rise bread, where it reached 10% relative abundance. Our results indicate that many of the foods prepared and consumed during the workshop were rich sources of probiotic microbes.

In contrast to the lactic acid ferments, others including the later koji-based ferments, tempeh, goat milk, kefir chèvre, and sweet potato fly, had large contributions from other genera that are commonly associated with food spoilage and food-borne pathogenicity (e.g., *Acinetobacter*, *Bacillus*, *Yersinia*, and *Pseudomonas*) [87–91]. In the most extreme example of a pathogenic member of these genera *Y. pestis*—which we thankfully did not observe in any of the samples we collected and consumed at the workshop—is the microbe responsible for plague [92]; however, other species of *Yersinia *can have more benign effects. Strains of *Yersinia* have been reported in traditional and dairy products in Iran [90], and both genera have been linked to a “fruity off flavour” in milk [91]. Species of *Acinetobacter*, including *A. baumannii*, which we observed at highest abundance in some of the later-stage koji-based ferments, have been associated with a large number of maladies and often occur in clinical settings with poor sanitation [88]. In some cases (e.g., tempeh and salt-rise bread), the cooking process likely rendered most bacterial strains inert. In others, such as miso, continued fermentation will likely result in substantial changes in the microbial community. Nonetheless, many of these “pathogenic” microbes are likely benign strains of genera and families often used as indicators of non-sterile sanitation practices.

A limitation of our study is that we only sought to identify bacterial taxa. Furthermore, the 16S metabarcoding approach we used can rarely identify bacteria beyond the genus or species level. Determining precisely which bacterial (and fungal) strains are present in these fermented foods—and potentially their functional and health consequences—would be aided greatly by metagenomic assembly and metabolomic approaches. Ferments like koji and tempeh are dominated by fungi (typically *Aspergillus oryzae* and *Rhizopus* spp., respectively), which could interact with bacteria we observed. To similar effect, after longer periods of fermentation, the sake and sweet potato fly will develop alcohol, which will introduce a new selective environment. Nonetheless, it is worth noting that inoculated fungal ferments can have rich bacterial communities that result from wild fermentation. Additionally, our study only sampled a small number of food items. Broader scale replication within these food items as well as expansion to other production environments would greatly improve our ability to understand the variation within fermented foods and the consequences of their consumption.

## Conclusion: linking social and biological cultures

Ultimately, we are interested in how stories and experiences with fermentation create different microbial possibilities. The fermenters who celebrate the non-homogenous tastes and practices that go along with these foods will be happy to see the wide range of variation in bacterial genera that accompany different categories of fermented foods. Interestingly, while different broad categories of foods had similar overall microbiological communities, like dairy, grain-based, or salted vegetable ferments, particular foods within these categories, like injera vs. dosa or kimchi vs. sauerkraut, varied in measurable ways, suggesting that small variations in food preparation may have a significant impact on the resulting microbiota even when these are prepared in the same kitchens. This was especially true for pao cai, a fermented vegetable dish involving regular infusions of new ingredients. Through these small variations in preparations, home fermentation appears to foster a wide range of microbial landscapes where a variable set of microbes can thrive. Subtle variations in “funk,” as Carl described these flavors above, are a consequence of these different sets of similar landscapes. This analysis shows that home ferments do indeed provide a mechanism for a more personalized, micro-biodiverse meal than would be possible when eating the kind of homogenized foods scorned by Warren in his critique of a “Walmartization” of flavor. This may be contrasted to many commercial fermented foods, which are sterilized and then inoculated with specific bacterial strains to provide a uniformity of taste and consistency in regulatory approval. One home-fermented exception appears to be yogurt, which experienced relatively little microbial change as a result of local variations in the circumstances of its fermentation. Instead of microbial change over time, the initial heating of the milk and inoculating bacterial culture takes on a special importance in shaping the resulting microbiota in this case.

Recalling Paxson’s [25] post-Pasteurian analysis of microbes as having a range of potential impacts including beneficial, harmful, and neutral, our analysis showed evidence of many different microbial genera. It is clear that the fermented foods produced and consumed during the workshop were a rich source of lactic acid bacteria, which we expected to find. Our results are consistent with the notion that food fermentation involves a wide variety of microbes, each of which has strain-specific effects that should not be predetermined based upon the known effects of conspecific or congeneric microbes. The 25 ASVs we identified as having potentially transmitted from fermented foods to the guts of the workshop participants are largely consistent with the range of microbes found in the food samples; 12 of them are lactic acid bacteria, and several others are from genera commonly found in foods (e.g., *Acinetobacter*, *Erwinia*, *Bacillus*). Given the lack of strain-specific and controlled health data, it is difficult to ascertain whether or not some of the ASVs we identified do in fact have negative or positive health impacts. For example, we observed the transmission of *Bacillus cereus*. Remarkably, some strains of *Bacillus cereus* are used as probiotics, while others are known to cause an anthrax-like disease [93–95]. Furthermore, given the potential risks of consuming *Yersinia* and *Pseduomonas*, one might question the wisdom of consuming the goat milk kefir chèvre or sweet potato fly we produced. In spite of these clinical associations, no one in the workshop complained of ill health. On the contrary, most participants reported excellent digestive health. This speaks to the importance of analysis beyond the genus level, which can differentiate beneficial, neutral, and harmful bacteria of the same genera. Further research to assemble the full genomes of these microbes will not only allow for finer grained identification of bacterial strains, but also allow for the study of their metabolic functions.

The probiotic microbes we detected would likely be welcome and an affirming news to participants who stressed potential health benefits in consuming home-fermented foods. Further, our comparison of foods and stools suggests that, as in clinical studies referenced earlier, these bacteria impact gut ecology over, at least, short periods of time. It is beyond the scope of this study to speculate whether a gut microbiome could fundamentally shift as a result of regularly eating fermented foods. However, each participant clearly had their own homeostasis and moved within a distinct personal range throughout the course of the study. Given the differences in gut microbiome observed between populations of humans in other research and the 25 genera we identified as having likely crossed between foods and guts, it is reasonable that fermented foods consumed in situ as part of a regular diet would influence gut microbiome composition.

The idea that landscapes are culturally formed is familiar to ethnobiologists. In this paper, we have simply focused on these interactions on a different scale. As conceived by a community of enthusiastic fermentation revivalists during a 2018 workshop, fermentation offers a space to embrace creative tactile work, consider one’s own body as a site of microbial transformation, and build relationships to both food production and microbial communities seen as beneficial. Variations within similar foods prepared at the same site as well as variations observed between different kinds of food suggest that this creative work, whether part of a traditional heritage practice or a newly discovered craft, results in a wide range of microbial communities within and without. Further, we show that there is reason to believe that these foods can introduce potentially probiotic microbes to human gut microbiomes, with the potential to offer at least short-term health benefits.

In combining the methodologies of cultural anthropology with microbiology, we also illustrate the importance of multidisciplinary fieldwork to ask about the complex synergies of health, production, and place in the food system. Anthropological research is well poised to collect perceptions of foodways, record sensory data, and use ethnographic detail to uncover the subtle variations in recipes. However, it is not well-positioned to link those findings to microbiological outcomes or quantitatively measure microbiological differences. That is, quantitative microbiology can determine which microbes are present or passing between people and foods, while qualitative ethnographic can explain why those discrepancies might exist or how they become meaningful. Such multi-disciplinary perspectives on data collection, analysis, and interpretation are especially helpful in conducting studies like this, which are based *in situ* and thus face innumerable confounds in quantitatively linking variables as a result of variations in human behavior. From an ethnographic perspective, however, these confounds are simply normal life. This combination of approaches is critical to link craft fermentation to the microbiomes of people who eat fermented foods. This mixed methods biocultural research design also suggests the importance of consuming these probiotics as a component of a larger food system that draws on social and biological networks. Although collaborations between anthropologists and microbiologists is rare, such careful attention to the collection of both sociocultural data and biological samples is common in ethnobiology. We hope to build on this tradition even as we shrink the scale.

## Data Availability

Qualitative data in the form of transcripts have not been included, except as quotes or themes in the article text, because that information is not approved or consented to be shared in this way. Please contact the authors for additional data requests.
